# Supplementary figures and images for: Antinociceptive effect of Equisetum arvense extract on the stomatitis hamster model
Source: PLoS One. 2024 Nov 21;19(11):e0313747. doi: 10.1371/journal.pone.0313747 (PMC11581248; doi:10.1371/journal.pone.0313747)

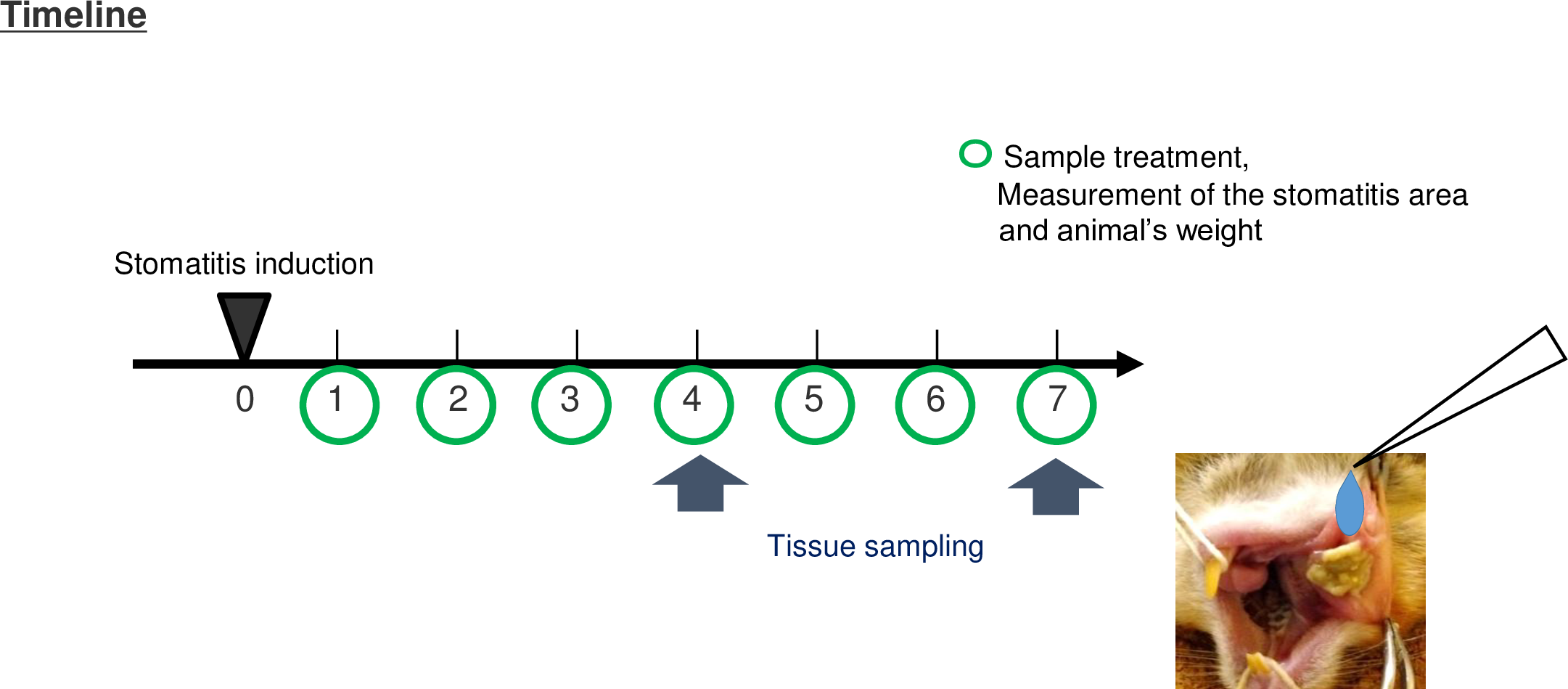

Supplement: S1 Fig — Starting from the day after stomatitis induction, 50 μL of EA (15 μg/mL or 1500 μg/mL) or Physiological saline (PS) was applied once daily for seven consecutive days, and the wound area was measured. The hamsters from each group were sacrificed 4th and 7th days after the intramucosal injection of acetic acid solution into the cheek pouch; the cheek pouches were collected, and the tissue sections were prepared for histological evaluation. (TIF) [file pone.0313747.s002.tif]
