# Supplementary material for: Antinociceptive effect of Equisetum arvense extract on the stomatitis hamster model
Source: PLoS One. 2024 Nov 21;19(11):e0313747. doi: 10.1371/journal.pone.0313747 (PMC11581248; doi:10.1371/journal.pone.0313747)
Supplement: Attachment — Submitted filename: Response_to_Reviewers.docx [file pone.0313747.s003.docx]

PONE-D-24-23705

Antinociceptive and healing-promoting effects of *Equisetum arvense* extract on the stomatitis hamster model

PLoS ONE

**Response to Reviewers**

We have responded to the reviewer's comments (blue) as follows. Please check.

**Reviewer #1:** The study design was appropriate to answer the research question (including the use of appropriate controls), and the conclusions supported by the evidence were presented.
The methods were sufficiently described to allow the study to be repeated.
The use of statistics and treatment of uncertainties were appropriate
The presentation of the work was clear.

Thank you for your positive feedback on our research from a specialist's perspective. Your comments have been a great encouragement to us. Thank you once again.

**Reviewer #2:** The manuscript entitled "Antinociceptive effect of Equisetum arvense extract on the stomatitis hamster model" was reviewed. Please respond to the questions and perform some corrections or changes suggested as follows
1. Why did you use Equisetum arvense extract especially for this research although there are many other medicinal plant extracts or synthetic materials with the same properties?
2. Why did you use the hamster model instead of the rat or mouse model?
3. Did you stain the pathological sections with histochemical stains such as Van Gieson or Masson's trichrome to compare collagen fibers in the groups?
4. Some figures have no scale number in the histopathologic figure (figure 2). That's better the number of the scales to be written in black color.
5. Considering each plant's extract consists of different components, do you think which EA extract especially has the potential ability to reduce pain and promote wound healing? Accordingly, do you think it is not better to purify the main components of this herbal extract and use the more useful components of this extract?
6. Why didn't you use different concentrations of EA in this research comparatively?
7. Considering that in the first days of wound formation, cells with neutrophils predominate and given the source of Cox 2 production is mostly monocytes, macrophages, and fibroblasts, don't you think measuring Cox 2 on days 4 and 7 in addition to 10 h after creating wound could be useful for a more accurate evaluation? However, pain reduction in the first 24 hours can be important for feeding.
8. What is the unit of SP content that you measured by ELISA? You mentioned the ratio in figure 5.
9. The measuring time of SP content is not mentioned in the text. SP modulates chronic pain and inflammation. So why did you measure it for acute pain? Wouldn't it be better if you measured and compared it on different days?

Thank you for your valuable, accurate, and critical comments. I have responded to them in bullet points as follows.

1. Thank you for your important question. For the following reasons, we tried to verify the effects of EA in this study.

The genus Equisetum is widely distributed worldwide and is only absent in Australasia and Antarctica (1, 2). Therefore, EA is easy to obtain as a natural plant resource (Consequently, it is not rare and can be obtained at a low price). Furthermore, it is often used in traditional medicine with diverse applications in many countries; therefore, several people are familiar with it and have no resistance to using it.

1. Calvo MI, Cavero RY. Medicinal plants used for cardiovascular diseases in Navarra and their validation from Official sources. J Ethnopharmacol. 2014;157: 268–273. doi: 10.1016/j.jep.2014.09.047.
2. Wright CI, Van-Buren L, Kroner CI, Koning MM. Herbal medicines as diuretics: a review of the scientific evidence. J Ethnopharmacol. 2007;114: 1–31. doi: 10.1016/j.jep.2007.07.023.
2. Thank you for your critical comment.　We used hamsters for the following reasons.
4. The golden Syrian hamster (*Mesocricetus auratus*) has long been a valuable rodent model for human diseases, especially stomatitis, due to its buccal mucosa, which is suitable for stomatitis models, and there are many previous studies on stomatitis using hamsters. (1, 2)
5. *Mesocricetus auratus* is a better model for immunopathological research than mice and rats and is used as a model for various diseases. (3-11)

The use of hamster models is an essential element of this research; therefore, we have mentioned this in the discussion section.

1. Katayama S, Nishizawa K, Hirano M, Yamamura S, Momose Y. Effect of polaprezinc on healing of acetic acid-induced stomatitis in hamsters. J Pharm Pharm Sci. 2000;3: 114–117.
2. Kadowaki R, Ogata F, Nishida M, Komatsu M, Otake H, Nakazawa Y, et al. Therapeutic effects of hydrogel formulations incorporating Troxipide nanoparticles on oral mucositis in Hamsters. Drug Des Devel Ther. 2023;17: 3349–3361. doi: 10.2147/DDDT.S433776.
3. Phillips LM, Li S, Gumin J, Daou M, Ledbetter D, Yang J, et al. An immune-competent, replication-permissive Syrian Hamster glioma model for evaluating Delta-24-RGD oncolytic adenovirus. Neuro Oncol. 2021;23: 1911–1921. doi: 10.1093/neuonc/noab128.
4. Li Z, Xiong C, Mo S, Tian H, Yu M, Mao T, et al. Comprehensive transcriptome analyses of the fructose-fed Syrian Golden hamster liver provides novel insights into lipid metabolism. PLoS One. 2016;11: e0162402. doi: 10.1371/journal.pone.0162402.
5. Ebihara H, Zivcec M, Gardner D, Falzarano D, LaCasse R, Rosenke R, et al. A Syrian golden hamster model recapitulating ebola hemorrhagic fever. J. Infect. Dis. 2013;207: 306–318. doi: 10.1093/infdis/jis626.
6. Guillaume V, Wong KT, Looi RY, Georges-Courbot MC, Barrot L, Buckland R, et al. Acute Hendra virus infection: Analysis of the pathogenesis and passive antibody protection in the hamster model. Virology. 2009;387: 459–465. doi: 10.1016/j.virol.2009.03.001.
7. Jove M, Ayala V, Ramirez-Nunez O, Serrano JC, Cassanye A, Arola L, et al. Lipidomic and metabolomic analyses reveal potential plasma biomarkers of early atheromatous plaque formation in hamsters. Cardiovasc. Res. 2013;97: 642–652. doi: 10.1093/cvr/cvs368.
8. Paciello O, Wojcik S, Gradoni L, Oliva G, Trapani F, Iovane V, et al. Syrian hamster infected with Leishmania infantum: A new experimental model for inflammatory myopathies. Muscle Nerve. 2010;41: 355–361. doi: 10.1002/mus.21502.
9. Best EL, Freeman J, Wilcox MH. Models for the study of Clostridium difficile infection. Gut Microbes. 2012;3: 145–167. doi: 10.4161/gmic.19526.
10. Wahl-Jensen V, Bollinger L, Safronetz D, de Kok-Mercado F, Scott DP, Ebihara H. Use of the Syrian hamster as a new model of ebola virus disease and other viral hemorrhagic fevers. Viruses. 2012;4: 3754–3784. doi: 10.3390/v4123754.
11. Safronetz D, Ebihara H, Feldmann H, Hooper JW. The Syrian hamster model of hantavirus pulmonary syndrome. Antivir Res. 2012;95: 282–292. doi: 10.1016/j.antiviral.2012.06.002.
3. We stained the pathological sections with Masson's trichrome to compare collagen fibers in the groups (Fig 3). Thank you for your wonderful suggestions that have helped improve our research.

4. Thank you for your accurate comments. All histopathological diagrams have been changed to include the following scale and scale number (Figs 2 and 3).

5. Thank you for giving us a logical realization. We believe that the healing-promoting and pain-relieving effects of EA are due to the quercetin it contains. For example, quercetin promotes wound healing through the extracellular signal-regulated kinase 1/2 mitogen-activated protein kinase and nuclear factor kappa B pathways (1). In addition, quercetin suppresses SP expression in the peripheral blood and conjunctival tissue of a mouse model of allergic conjunctivitis (1), and quercetin reportedly inhibits p30 0 signaling and COX-2 expression by blocking the binding of multiple transcription factors to the COX-2 promoter in vitro (2). Therefore, we hypothesized that it would be better to use purified quercetin or EA, which contains a high amount of quercetin. We believe these points were very crucial; therefore, we have included them in the main text as future research prospects.
1. Burcin Beken, Riza Serttas, Mehtap Yazicioglu, Kader Turkekul, Suat Erdogan. Quercetin Improves inflammation, oxidative stress, and impaired wound healing in atopic dermatitis model of human keratinocytes. Pediatr Allergy Immunol Pulmonol. 2020;33: 69–79. doi: 10.1089/ped.2019.1137.
2. Ding Y, Li C, Zhang Y, Ma P, Zhao T, Che D, et al. Quercetin as a Lyn kinase inhibitor inhibits IgE-mediated allergic conjunctivitis. Food Chem Toxicol. 2020;135: 110924. doi: 10.1016/j.fct.2019.110924.
3. Xiao X, Shi D, Liu L, Wang J, Xie X, Kang T, et al. Quercetin suppresses cyclooxygenase-2 expression and angiogenesis through inactivation of P300 signaling. PloS One. 2011;6: e22934. doi: 10.1371/journal.pone.0022934.
6. Thank you for your excellent advice. We believe your advice was a very critical point; therefore, we conducted a similar test using EA at a concentration 100 times stronger and verified the change in the effect of EA due to the difference in concentration. Thus, when the EA concentration was increased 100 times, a healing-promoting effect was confirmed (Figs 1-4). Please check the main text for details. Based on this, the title of the manuscript has been revised to Antinociceptive and Healing-promoting effects of Equisetum arvense extract on the stomatitis hamster model.
7. Thank you for pointing this out. The figure provided below presents the preliminary data (n = 2). An examination of the expression of COX-2 mRNA using quantitative reverse transcription polymerase chain reaction in the wound tissue over time from the formation of the wound showed that COX-2 mRNA increased the most after 10 h; therefore, we decided to evaluate it at that point.

8. Thank you for your valuable question. We used the cultured dorsal root ganglion cells which contain primary sensory neurons. The average content of SP spontaneously released from the cultured cells in the incubation medium for 10 min was 480 pg/mL when measured by ELISA. The calculation method for the Substance P release ratio has been added to the materials and methods section.
9. Thank you for your comment. SP also acts as a substance for acute pain and exerts several immunological actions. For example, SP participates in the migration of several innate immune cells, such as neutrophils (1).　Finally SP modulates the inflammation events resulting in the sensation of chronic pain. In this experiment, the SP released from the cultured dorsal root ganglion cells in the incubation medium for 10 min was measured and the inhibiting effects of *Equisetum arvense* extract on the releasing pathway of SP have been shown. We added the incubation time, 10 min in the text, which is also drawn in the “Methods”.
1. Serra MC, Calzetti F, Ceska M, Cassatella MA. Effect of substance P on superoxide anion and IL-8 production by human PMNL. Immunology. 1994;82: 63–69.

**Reviewer #3:** Shiba F et al., showed the interesting study on the “Antinociceptive effect of Equisetum arvense extract on the stomatitis hamster model”. The results are drastically significant and study designs are interesting; however, authors need some additional modifications. Some concerns below needs to be addressed.
1- Page 3, line 66, it should be anti-osteoclastogenesis.
2- Did authors check the effect of EA on other inflammatory mediators?
3- Figure 3, why Day4’s ratio of [Weight Gain %] of stomatitis/EA vs stomatitis/PS is higher than others? Did authors perform statistical analysis on Day2-6 of stomatitis/EA vs stomatitis/PS?
4- Bdkrb1appeared in the manuscript. Can authors discuss more why Bdkrb1 expression was not affected?
5- To make this manuscript potential enough and unique in term of “Promotion of Wound Healing” authors should discuss more about how liquid EA application is different from ointment.

Thank you for your valuable, accurate, and critical comments. I have responded to them in bullet points as follows.

1. Thank you for pointing this out.　On page 4, line 69, it has been corrected to anti-osteoclastogenesis.
2. Thank you for your great comment. Following your comment, we also verified the mRNA expression of IL-1β and IL-8 as other inflammatory mediators; however, we could not obtain these data. The fact that we could not evaluate a wide range of inflammatory mediators has been presented as a limitation of the study.
3. Thank you for your good question. We conducted statistical analysis between all counties on days 1-6. Based on your comment, the ratio of [Weight Gain %] of stomatitis/EA vs stomatitis/PS on day 4 is higher than that on other days; however, this is considered due to individual differences within the same group. Furthermore, no statistically significant difference was observed (Fig 4).
4. Thank you for your valuable comments. At the 10-h point after inducing stomatitis, the mRNA expression level of Bdkrb1 was not significantly higher in the Stomatitis/PS group than in the Healthy group, suggesting that the timing of tissue collection was not optimized. This is one of the limitations of the study, and we have described this in the discussion section.
5. Thank you for your excellent advice. We evaluated the 100-fold concentrated EA (Stomatitis/100*EA) similarly based on the comments from #Reviewer 2. Therefore, we confirmed a significant healing-promoting effect of EA. We decided not to mention the difference in effectiveness between the ointment and liquid preparations in the main text; however, we believe that the ointment preparation is more effective than the liquid preparation based on the following reasons.

Stomatitis is a condition in which the surface of the wound becomes ulcerated; therefore, it is expected that the ointment preparation is more likely to promote moist wound healing than a liquid formulation at the same EA concentration. Furthermore, EA retention will be enhanced and more easily absorbed through the oral mucosa. Many reports exists on the healing-promoting effects of EA ointment (1, 2, 3); however, there are no reports on EA liquid.

1. Ozay Y, Kasim Cayci M, Guzel-Ozay S, Cimbiz A, Gurlek-Olgun E, Sabri Ozyurt M. Effects of Equisetum arvense ointment on diabetic wound healing in rats. Wounds. 2013;25: 234–241.
2. Ozay Y, Ozyurt S, Guzel S, Cimbiz A, Olgun EG, Cayci MK. Effects of Equisetum arvense ointment on dermal wound healing in rats. Wounds. 2010;22: 261–267.
3. Asgharikhatooni A, Bani S, Hasanpoor S, Mohammad Alizade S, Javadzadeh Y. The effect of equisetum arvense (horse tail) ointment on wound healing and pain intensity after episiotomy: a randomized placebo-controlled trial. Iran Red Crescent Med J. 2015;17: e25637. doi: 10.5812/ircmj.25637
